# Non-traumatic vitreous base detachment in an adult with neurofibromatosis type 1: a case report

**DOI:** 10.1016/j.ajoc.2026.102697

**Published:** 2026-10-26

**Authors:** Hassan Asadigandomani, Xinyi Chen, Jay M. Stewart, Rabih Hage, Frank Brodie

**Affiliations:** a Department of Ophthalmology, University of California San Francisco, San Francisco, CA, 94143-4081, USA; b Department of Surgery, San Francisco Veterans Affairs Hospital, San Francisco, CA, USA

**Keywords:** Neurofibromatosis type 1, Vitreous base detachment, Vitreoretinal traction, Optic neuropathy, Case report

## Abstract

**Purpose:**

Although neurofibromatosis type 1 (NF1) is best known for its optic pathway and anterior segment manifestations, posterior segment involvement can occur. Vitreoretinal traction and retinal detachment (RD) have been most commonly reported, but spontaneous vitreous base detachment has been only infrequently reported.

**Observations:**

We describe a 33-year-old woman with NF1 in whom multimodal retinal imaging of the right eye revealed a well-demarcated vitreous base detachment with peripheral tractional RD, nasal vascular tortuosity, and no evidence of retinal breaks or active inflammation, in the absence of any history of ocular or head trauma. The tractional changes remained stable during follow-up, and no intervention was required.

**Conclusions and importance:**

This case highlights an unusual posterior segment manifestation of NF1 and underscores the importance of careful peripheral retinal examination, with consideration of wide-field imaging when clinically indicated, during the ophthalmic follow-up of adults with NF1.

## 1 Introduction

Neurofibromatosis type 1 (NF1) is a hereditary neurocutaneous disorder transmitted in an autosomal-dominant pattern and caused by pathogenic variants in the *NF1* tumor suppressor gene.^1^ Ocular findings are common, particularly optic pathway gliomas, iris Lisch nodules, and choroidal abnormalities, all of which are included in the revised diagnostic criteria for NF1.^1^, ^2^, ^3^ Standard ophthalmic surveillance in NF1 primarily focuses on identifying optic pathway gliomas during childhood.^4^

NF1 can also involve the retina, although retinal findings are not part of the diagnostic criteria.^5^, ^6^ Recent reports suggest that individuals with NF1 may also develop retinal complications, including retinal detachment (RD), during adolescence or adulthood.^7^, ^8^, ^9^, ^10^ Within this spectrum of retinal disease, vitreoretinal tractional abnormalities appear to be relatively uncommon, and only a few cases of spontaneous vitreous base detachment have been reported in association with NF1.^11^, ^12^

In this report, we present an NF1 patient with chronic peripheral vitreoretinal traction characterized by a well-defined spontaneous vitreous base detachment.

## 2 Case presentation

A 33-year-old woman with a history of NF1 initially presented with progressive, painless visual decline in the left eye. Neuroimaging demonstrated T2/FLAIR hyperintensity of the left optic nerve, initially raising concern for optic pathway glioma. However, serial magnetic resonance imaging (MRI) showed interval improvement of the signal abnormality, favoring an inflammatory optic neuropathy over a neoplastic process. An extensive neuro-ophthalmic evaluation, including investigation for autoimmune and inflammatory etiologies in conjunction with neurology, did not identify an alternative systemic diagnosis. The patient was treated with repeated courses of high-dose corticosteroids and subsequently underwent plasmapheresis because of progressive, steroid-refractory vision loss in the left eye. Although the left eye ultimately progressed to profound visual loss, the patient remained under close neuro-ophthalmic surveillance.

On ophthalmic examination, the left eye demonstrated no light perception, a relative afferent pupillary defect (RAPD), and optic disc pallor. Optical coherence tomography (OCT) revealed marked thinning of the retinal nerve fiber layer and ganglion cell complex, while the macula remained structurally intact. The anterior segment was otherwise unremarkable in both eyes, with no evidence of anterior chamber inflammation, keratic precipitates, or posterior synechiae. The right eye had preserved visual acuity, a normal-appearing optic disc, and an unremarkable anterior segment. During follow-up for the left-eye optic neuropathy, subtle functional abnormalities were detected in the fellow (right) eye, prompting retinal evaluation, during which the vitreoretinal abnormalities described below were incidentally identified. Wide-field fundus photography revealed a well-demarcated vitreous base detachment extending from 8:00 to 11:00, together with a shallow inferonasal tractional RD from 4:00 to 6:00, consistent with chronic vitreoretinal traction (Fig. 1). Subsequent ultrasonography confirmed a shallow inferior RD with a taut peripheral vitreous band.

**Fig. 1:**
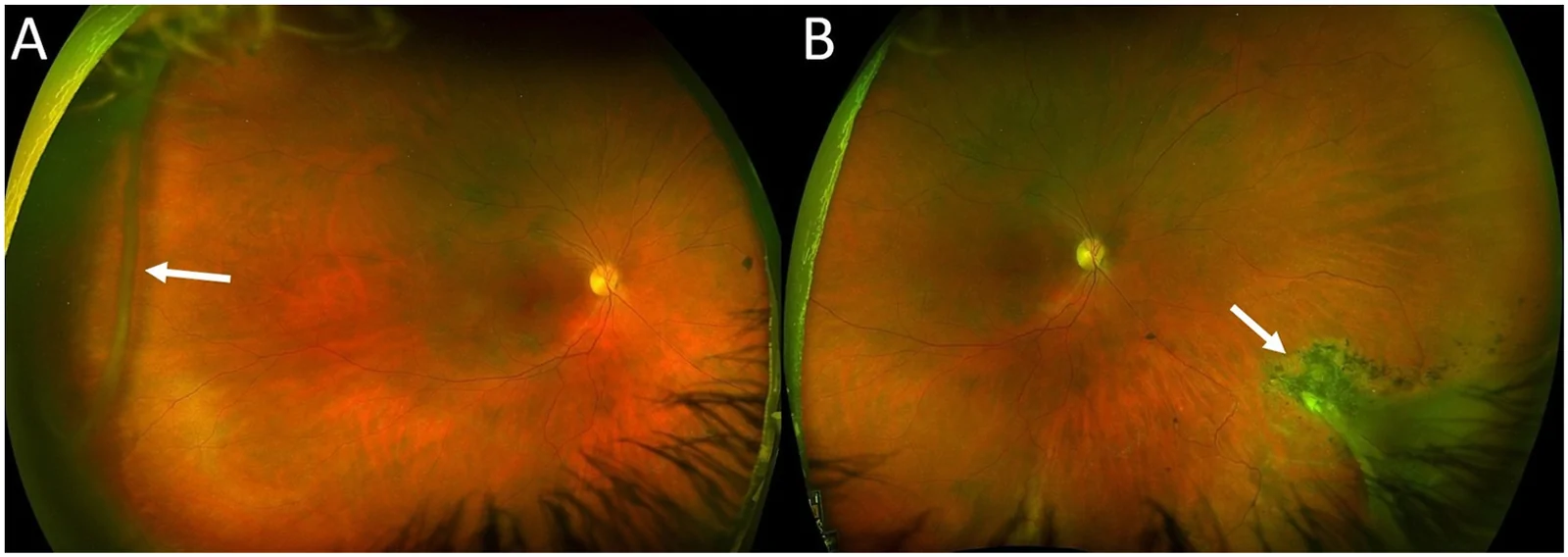
Wide-field fundus photograph of right eye (A) showing vitreous base detachment from 8:00 to 11:00, and (white arrow) (B) peripheral tractional RD at 4:00–6:00 (white arrow) with associated vitreous base traction and nasal vascular tortuosity.

Fluorescein angiography (FA) showed focal microvascular abnormalities without peripheral non-perfusion, vasculitis, or leakage suggestive of active inflammation. Indocyanine green angiography (ICGA) was normal bilaterally, effectively ruling out choroidal neurofibromas (Fig. 2).

**Fig. 2:**
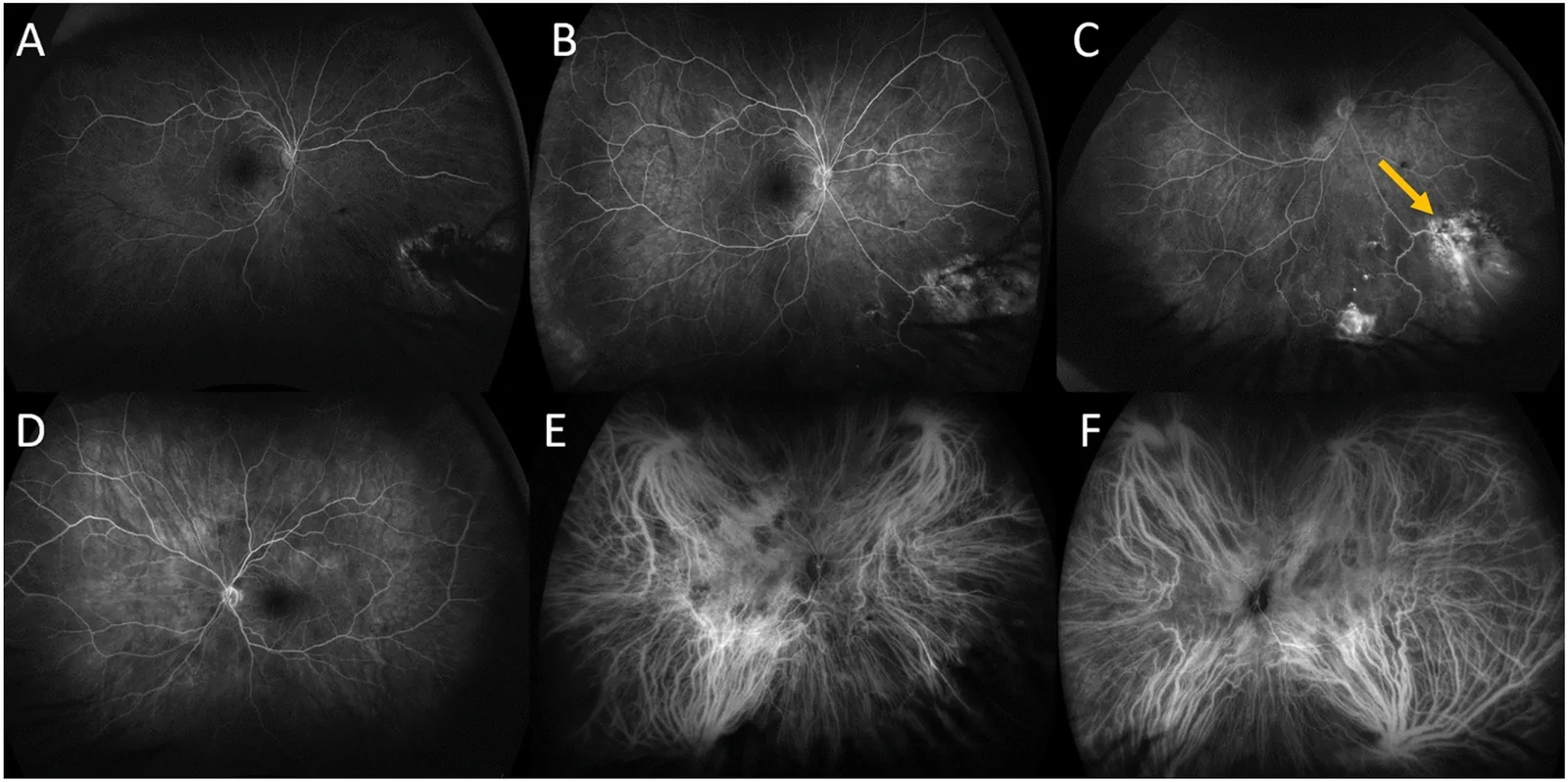
Wide-field FA of the right eye (A–C) demonstrating normal retinal perfusion without leakage, vasculitis, or peripheral non-perfusion, consistent with a chronic, non-inflammatory tractional process (yellow arrow). Wide-field FA of the left eye (D) is unremarkable. Wide-field ICGA of the right eye (E) and left eye (F) reveals no choroidal abnormalities. (For interpretation of the references to colour in this figure legend, the reader is referred to the Web version of this article.)

Near-infrared reflectance imaging of the right eye demonstrated focal hyper-reflective choroidal nodules with corresponding hyperreflective lesions on OCT (Fig. 3). Such nodules have been reported with high prevalence in NF1 and represent an established posterior segment finding, even in asymptomatic patients.

**Fig. 3:**
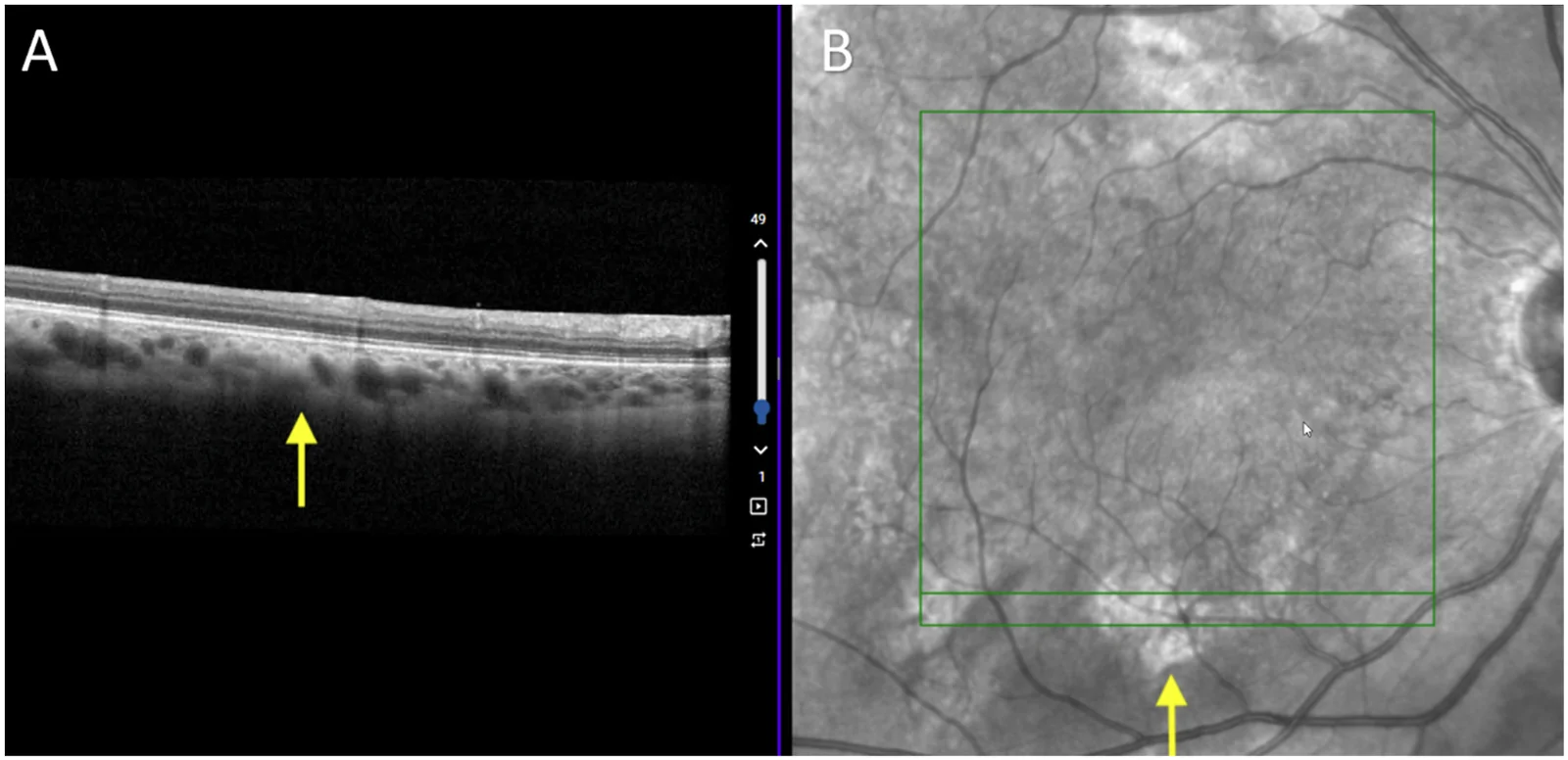
OCT (A) showing a focal hyperreflective choroidal lesion (yellow arrow), with a corresponding hyper-reflective choroidal nodule on near-infrared reflectance imaging (B) (yellow arrow). (For interpretation of the references to colour in this figure legend, the reader is referred to the Web version of this article.)

These findings were interpreted as chronic tractional pathology, possibly related to NF1-associated vitreoretinal abnormalities or longstanding microvascular changes. The presence of a peripheral vitreous base detachment in the right eye, in the absence of any history of ocular or head trauma, raised the possibility of an association with NF1. Serial wide-field imaging demonstrated no progression in RD or traction, and the detached vitreous base maintained a stable configuration over time. The lack of new hemorrhage, exudation, or retinal breaks strongly suggested a chronic, mechanically stable process rather than an active or progressive detachment.

The patient remains under combined neuro-ophthalmic and vitreoretinal follow-up. Because the peripheral tractional changes remained stable on serial examinations without retinal breaks or progression of the RD, observation was considered the most appropriate management strategy. Barrier laser or scleral buckle would be considered if progression of the tractional changes or RD were documented. Despite the irreversible loss of vision in the left eye, right eye function remains stable, supported by ongoing multimodal evaluations including OCT, wide-field imaging, FA, and ICGA.

## 3 Discussion

Spontaneous vitreous base detachment is an uncommon clinical finding and has historically been considered a hallmark of prior blunt ocular trauma.^13^, ^14^ However, emerging reports suggest that in individuals with NF1, structural abnormalities at the vitreoretinal interface may predispose to non-traumatic detachment of the vitreous base.^11^, ^12^ Although NF1 is classically known for optic pathway gliomas and cutaneous neurofibromas, an expanding body of literature highlights a broader range of posterior segment manifestations, including choroidal nodules, retinal vascular alterations, and, in rare cases, vitreoretinal tractional changes.^11^, ^12^, ^15^, ^16^, ^17^, ^18^ The current case contributes to the limited literature by documenting a unilateral, chronic-appearing vitreous base detachment in a patient with NF1 without a history of ocular trauma, further expanding the limited number of reported cases.

Ocular manifestations in NF1 primarily reflect dysfunction of neural crest–derived tissues. Although anterior segment findings are common, posterior segment abnormalities (including choroidal nodules and retinal vascular alterations) may be underrecognized.^17^, ^18^, ^19^ The rarity of vitreoretinal interface abnormalities in NF1 may relate less to true absence and more to limited peripheral retinal examination in asymptomatic adults, especially given that ophthalmic surveillance typically decreases after adolescence. This is clinically important because abnormalities of the vitreous base may remain silent for long periods yet predispose to retinal dialysis, tractional elevations, or rhegmatogenous RD (RRD).^7^, ^8^, ^9^

A possible mechanism underlying spontaneous vitreous base detachment in NF1 involves abnormalities of the vitreoretinal interface. Experimental studies have shown that neurofibromin-deficient fibroblasts exhibit altered proliferation and extracellular matrix organization, which could affect collagen architecture at the vitreous base.^12^, ^20^, ^21^, ^22^ Such mechanisms may provide a plausible biological explanation for the findings observed in the present case, although a causal relationship cannot be established.

A report by Desai documented unilateral temporal vitreous base detachment in a 49-year-old woman, associated with cobblestone degeneration but without retinal breaks or trauma.^12^ Another report by Jordan et al. described bilateral temporal detachments in a 40-year-old woman with NF1, again in the absence of a traumatic history.^11^ Compared with these cases, our patient demonstrated several distinguishing features: (1) a younger age at presentation and (2) the presence of associated tractional RD. Table 1 summarizes the similarities and distinctions among the published cases and underscores the potential variability of presentation across NF1 patients. Although the cases differed in laterality, refractive status, presenting symptoms, and associated retinal findings, all occurred in the absence of ocular trauma and demonstrated a stable clinical course during follow-up.

**Table 1:** Reported cases of spontaneous vitreous base detachment in patients with NF1.

Feature	Desai (1995)^15^	Jordan et al. (2023)^14^	Present case (2025)
**Age/Gender**	49-year-old female	40-year-old female	33-year-old female
**Race**	White	White	White
**Laterality**	Unilateral (right eye)	Bilateral	Unilateral (right eye)
**Refractive error**	High myopia (SE −6.75 D)	Emmetropia	Emmetropia
**Location of vitreous base detachment**	Temporal	Temporal bilaterally	Superotemporal (8:00–11:00)
**Associated retinal findings**	Cobblestone degeneration; retina attached, no tears	Retina attached; no tears	Infero-nasal localized tractional RD; nasal vessel tortuosity
**Symptoms at presentation**	Floaters for 2 months	New-onset floaters (1 week) & photopsia	Asymptomatic; vitreous base detachment found incidentally
**Evidence of trauma**	None	None	None
**Anterior segment findings**	Lisch nodules	Normal	Normal
**Course/Follow-up**	Stable; vitreous base detachment margin split into two segments	Stable bilaterally; no RD	Stable over multiple visits; no RD progression

Several alternative etiologies for the vitreous base detachment were considered, including remote ocular trauma, retinal dialysis, and degenerative vitreoretinal changes. The opposing distribution of the vitreous base detachment and inferonasal tractional RD raises the theoretical possibility of a contrecoup-type mechanism associated with prior blunt ocular trauma. However, a traumatic etiology was considered less likely because the patient repeatedly denied any history of ocular or head trauma, and serial examinations revealed no associated vitreous hemorrhage or peripheral retinal breaks. Retinal dialysis was also considered unlikely because no retinal dialysis or associated peripheral retinal break was identified on serial examinations, while ultrasonography demonstrated a taut peripheral vitreous band supporting a tractional mechanism. Furthermore, degenerative vitreoretinal changes were considered less likely given the patient's relatively young age, absence of high myopia or other predisposing peripheral retinal degeneration, and stable appearance of the lesion during follow-up. Although these alternative etiologies cannot be definitively excluded, the overall clinical and imaging findings favor a chronic, non-traumatic vitreoretinal tractional process.

An important consideration in this case is whether the patient's left-eye inflammatory optic neuropathy and right-eye vitreoretinal abnormalities represent manifestations of a single disease process. Based on the available clinical and imaging findings, we believe these are most likely two distinct processes occurring in the same patient with NF1 rather than manifestations of a unified inflammatory disorder. The optic neuropathy demonstrated radiographic evolution and a clinical course consistent with inflammatory optic nerve disease, whereas the right eye showed no evidence of intraocular inflammation on clinical examination or FA. Instead, the vitreous base detachment and associated chronic tractional retinal changes remained stable over serial examinations and were more plausibly consistent with structural abnormalities of the vitreoretinal interface that have been reported in association with NF1.

Because vitreous base detachments may remain clinically silent, they are likely underdiagnosed. Previous reports of asymptomatic RD in patients with NF1 further support that subtle vitreoretinal abnormalities may escape detection during routine examinations.^7^, ^8^, ^9^ While prior reports have primarily emphasized rhegmatogenous mechanisms, the present case raises the possibility that abnormal vitreoretinal adhesion associated with NF1 may contribute to chronic tractional RD, potentially reflecting persistent vitreous base traction secondary to disordered collagen architecture.

In summary, the present case expands the limited literature describing spontaneous vitreous base detachment in patients with NF1. Although a causal relationship cannot be established, this case highlights that clinically significant peripheral vitreoretinal abnormalities may occur in adults with NF1 and remain asymptomatic until detected on careful examination. Therefore, comprehensive peripheral retinal assessment, including scleral indentation and wide-field multimodal imaging when clinically indicated, may be valuable in selected adult patients with NF1. Additional case reports are needed to clarify the prevalence and clinical significance of this association and to inform future surveillance strategies.

## CRediT authorship contribution statement

**Hassan Asadigandomani:** Writing – original draft, Methodology, Investigation, Formal analysis, Data curation. **Xinyi Chen:** Writing – review & editing, Validation, Investigation. **Jay M. Stewart:** Writing – review & editing, Supervision. **Rabih Hage:** Supervision, Investigation. **Frank Brodie:** Writing – review & editing, Validation, Supervision, Formal analysis, Data curation, Conceptualization.

## Ethics declaration

This study was performed in compliance with relevant laws, regulatory frameworks and guidelines where the research took place. Institutional review board approval was not required for this case report per institutional policy. The report adheres to the tenets of the Declaration of Helsinki and was conducted in compliance with the Health Insurance Portability and Accountability Act (HIPAA). Written informed consent for publication of this report and any accompanying images was obtained from the patient.

This research follows the CARE guidelines and the CARE checklist.

## Availability of data and material

All clinical data supporting the conclusions of this case report are contained within the article.

## Artificial intelligence statement

The authors used ChatGPT (OpenAI) solely to assist with language editing and improvement of grammar, clarity, and readability during manuscript preparation. The authors reviewed and edited all AI-assisted text, verified the accuracy of the scientific content, and take full responsibility for the final version of the manuscript.

## Funding

There were no specific funding sources for this study.

## Declaration of competing interest

The authors declare that they have no known competing financial interests or personal relationships that could have appeared to influence the work reported in this paper.
